# P256: Survey of prevalence of healthcare associated infection in Chuyo Ouagadougou (Burkina Faso)

**DOI:** 10.1186/2047-2994-2-S1-P256

**Published:** 2013-06-20

**Authors:** J Zoungrana, A Traore, L Ouedraogo

**Affiliations:** 1 Hospital Hygiene Service, Centre Hospitalier Universitaire de Yalgado , Ouagadougou, Burkina Faso

## Introduction

Nosocomial infections (NI) are now a major public health problem that is insufficiently known and poorly mastered in our health facilities. The management of these infections is part of a comprehensive approach to improving the safety and quality of patient care.

## Objectives

To measure a point prevalence and describe the characteristics of nosocomial infections at the university hospital Yalagado and to analyze the anti-infective therapies.

## Methods

First prevalence survey of nosocomial infections (NI) in two departments of medicine and three surgical services performed using a standardized questionnaire with data collection approved by department heads. The five services represent 40.46% of all service beds in the institution (295 beds) and 114 patients were included in the study.

## Definition criteria

An infection is known as nosocomial if it was absent in a period of at least 48 h after admission to the hospital. Surgical site infections, were classified as nosocomial if infections occurred within 30 days following the surgery, or, in the year following the intervention in the case of the implementation of a prosthesis or implant.

## Results

On the day of the survey, 27 of 114 patients were infected resulting in a prevalence of 23.7%. In addition, x patients were treated with anti-infective therapy is a prevalence of x% . Three locations accounted for 77.79% of NI: urinary tract infection (14.82%), respiratory infection (18.52%), surgical site infection (44.45%). The prevalence of nosocomial infections varied according to the type of service (higher in surgery and intensive care) and patient characteristics.

## Conclusion

Surveillance of nosocomial infections is a good way to educate health workers and to draw their attention to the local infectious epidemiology, allowing the development of preventive actions at the services.

## Disclosure of interest

None declared

